# Hep‐CORE: a cross‐sectional study of the viral hepatitis policy environment reported by patient groups in 25 European countries in 2016 and 2017

**DOI:** 10.1002/jia2.25052

**Published:** 2018-04-10

**Authors:** Jeffrey V Lazarus, Samya R Stumo, Magdalena Harris, Greet Hendrickx, Kristina L Hetherington, Mojca Maticic, Marie Jauffret‐Roustide, Joan Tallada, Kaarlo Simojoki, Tatjana Reic, Kelly Safreed‐Harmon, the Hep‐CORE Study Group

**Affiliations:** ^1^ Barcelona Institute for Global Health (ISGlobal) Hospital Clínic University of Barcelona Barcelona Spain; ^2^ CHIP, Rigshospitalet University of Copenhagen Copenhagen Denmark; ^3^ Department of Social and Environmental Health Research London School of Hygiene and Tropical Medicine London United Kingdom; ^4^ Viral Hepatitis Prevention Board Centre for the Evaluation of Vaccination, Vaccine and Infectious Diseases Institute University of Antwerp Antwerp Belgium; ^5^ Clinic for Infectious Diseases and Febrile Illnesses University Medical Centre Ljubljana Ljubljana Slovenia; ^6^ Faculty of Medicine University of Ljubljana Ljubljana Slovenia; ^7^ Cermes3 (Inserm U988 UMR CNRS 8211 EHESS) Paris Descartes University Paris France; ^8^ European AIDS Treatment Group Brussels Belgium; ^9^ A‐Clinic Foundation Helsinki Finland; ^10^ European Liver Patients’ Association Brussels Belgium

**Keywords:** coinfection, drug therapy, health policy, hepatitis B, hepatitis C, viral hepatitis prevention and control

## Abstract

**Introduction:**

The first World Health Organization (WHO) global health sector strategy on hepatitis B and C viruses (HBV and HCV) has called for the elimination of viral hepatitis as a major public health threat by 2030. This study assesses policies and programmes in support of elimination efforts as reported by patient groups in Europe.

**Methods:**

In 2016 and 2017, hepatitis patient groups in 25 European countries participated in a cross‐sectional survey about their countries’ policy responses to HBV and HCV. The English‐language survey addressed overall national response; public awareness/engagement; disease monitoring; prevention; testing/diagnosis; clinical assessment; and treatment. We performed a descriptive analysis of data and compared 2016 and 2017 findings.

**Results:**

In 2017, 72% and 52% of the 25 European study countries were reported to not have national HBV and HCV strategies respectively. The number of respondents indicating that their governments collaborated with civil society on viral hepatitis control increased from 13 in 2016 to 18 in 2017. In both 2016 and 2017, patient groups reported that 9 countries (36%) have disease registers for HBV and 11 (44%) have disease registers for HCV. The number of countries reported to have needle and syringe exchange programmes available in all parts of the country dropped from 10 (40%) in 2016 to 8 in 2017 (32%). In both 2016 and 2017, patient groups in 5 countries (20%) reported that HCV treatment is available in non‐hospital settings. From 2016 to 2017, the reported number of countries with no restrictions on access to direct‐acting antivirals for HCV increased from 3 (12%) to 7 (28%), and 5 fewer countries were reported to refuse treatment to people who are currently injecting drugs.

**Conclusions:**

The patient‐led Hep‐CORE study offers a unique perspective on the readiness of study countries to undertake comprehensive viral hepatitis elimination efforts. Viral hepatitis monitoring should be expanded to address policy issues more comprehensively and to incorporate civil society perspectives, as is the case with global HIV monitoring. Policy components should also be explicitly added to the WHO framework for monitoring country‐level progress against viral hepatitis.

## 1 Introduction

Viral hepatitis is a major public health problem, with more deaths annually attributable to this group of diseases than to HIV, malaria or tuberculosis 1. Hepatitis B virus (HBV) and hepatitis C virus (HCV) infections account for 47% and 48% of viral hepatitis mortality respectively 2. Both HBV and HCV infections can lead to cirrhosis and liver cancer, and most HBV‐ and HCV‐related deaths occur as a direct result of one of these two diseases 1. HBV and HCV together are estimated to account for almost 80% of deaths from liver cancer 3, 4, which was the third most common cause of cancer mortality in 2013 5.

In the WHO European Region an estimated 18.5 million people are chronically infected with HBV, and an estimated 15 million people are chronically infected with HCV 6, 7. The annual number of deaths from viral hepatitis‐related causes in the Region is thought to exceed 170,000 8. With HBV vaccination now widespread in younger age groups, HBV tends to be seen more in older Europeans and in migrants from countries with high HBV prevalence 8. Practices associated with unsafe injecting drug use are a major contributor to the HCV epidemic in the European Region. There are high levels of HCV transmission among HIV‐positive men who have sex with men, and healthcare‐related transmission of HCV continues to occur in some countries 9, 10. The recent introduction of highly effective direct‐acting antiviral (DAA) drugs has made HCV much easier and safer to cure, but concern has been raised about whether health systems in Europe and globally will be able to overcome the numerous barriers to scaling up DAA treatment 10, 11, 12.

In 2014, in response to increasing recognition of the large disease burden imposed by viral hepatitis, the World Health Assembly approved a resolution calling on WHO Member States to enact various viral hepatitis control measures. The same resolution charged WHO with examining “the feasibility of and strategies needed for the elimination of hepatitis B and hepatitis C with a view to potentially setting global targets” 13. In this policy context, the European Liver Patients’ Association (ELPA) published 43 key recommendations for European governments 14. ELPA subsequently commissioned the Hep‐CORE (“Hepatitis – Community, Opinion, Recommendations, Experts”) study to assess the extent to which these recommendations are being followed on a national level in European countries. As the Hep‐CORE study was being planned, other major policy developments occurred. In 2015, United Nations Member States included a commitment to combat viral hepatitis in the Sustainable Development Goals 15 and in 2016, WHO launched its first global health sector strategy on viral hepatitis 2. The strategy sets forth the ambitious targets of achieving a 90% reduction in new cases of chronic HBV and HCV and a 65% reduction in HBV and HCV deaths, all by 2030 2.

To reach the targets and achieve WHO's overarching goal of eliminating viral hepatitis as a major public health threat by 2030, governments around the world must first consider whether they have the necessary policies and programmes in place. Scant information is available regarding the current policy landscape. The 2013 *Global Policy Report on the Prevention and Control of Viral Hepatitis in WHO Member States* presents findings from a policy survey completed by representatives of 126 national governments 16. A follow‐up survey of civil society organizations found that numerous respondents questioned the accuracy of the information reported by their governments 17.

Policy monitoring of national responses to major health issues has been approached in a variety of ways 18, 19. A key example is the global HIV policy monitoring process coordinated by the Joint United Nations Programme on HIV/AIDS (UNAIDS) for more than a decade. Governments are asked to prepare periodic reports on their countries’ progress in combating HIV, with some of the information gathered using a survey known as the National Commitments and Policy Instrument (NCPI) 20. Significantly, one part of the NCPI is completed by governments and the other by in‐country civil society stakeholders.

With no ongoing government or multi‐stakeholder policy monitoring process for viral hepatitis in place at the global or regional level, ELPA sought to fill a gap by implementing the Hep‐CORE study in the countries where it had member organizations. These patient groups were responsible for reporting information to the study team. ELPA's goal was twofold: (1) to help liver patient groups identify key policy shortcomings in study countries; and (2) to engage liver patient groups in a regional policy monitoring initiative that will help hold countries accountable to their pledges to work towards viral hepatitis elimination. The following article reports findings from the 2016 and 2017 Hep‐CORE surveys.

## 2 Methods

A research team based at the University of Barcelona and the University of Copenhagen implemented the Hep‐CORE study in consultation with a multidisciplinary study group of viral hepatitis experts and carried out two rounds of data collection: one from July to October 2016 and another from August to November 2017.

The original study instrument, a 39‐item English‐language online survey (Additional File 1), reflected ELPA's recommendations to European governments 14. We developed and revised the survey in accordance with multiple rounds of input from study group members. The 2016 survey questions addressed issues relating to all seven categories of recommendations: overall national response, public awareness and engagement, monitoring and data collection, prevention, testing and diagnosis, clinical assessment, and treatment. The study instrument was piloted in June 2016. Four prospective study participants completed the survey and provided feedback, which guided final revisions. The Hep‐CORE 2017 study instrument comprised 11 questions derived from the 2016 survey (Additional File 2). We piloted it in August 2017 with five study group members. The pilot responses and additional feedback guided survey modifications.

As in 2016, the main survey questions in 2017 were closed‐ended, as were most sub‐questions. For both rounds of data collection, we asked study participants to complete the survey using Research Electronic Data Capture (REDCap), a web‐based data collection tool that enables responses to be saved and edited over the course of multiple sessions 21. The survey instructions asked respondents to conduct research as needed to answer survey questions accurately, and recommended contacting sources such as government officials and viral hepatitis experts. Respondents were able to pause work on their surveys and log back into them to change or add information using unique access codes.

The study cohort was recruited through a purposive sampling process. We emailed an invitation to participate in the survey to one liver patient group in each of the 24 European countries where ELPA had members at the time of 2016 study recruitment. In countries with more than one patient group, we selected the most representative group that was involved in viral hepatitis advocacy. We also emailed the invitation to a liver patient group in Denmark, since the patient group was just about to join ELPA, and did so in 2017. The same 25 patient groups that accepted the invitation and contributed data in 2016 were invited to respond to the 2017 survey. Many of the same individuals completed the survey in both years, although personnel changes in some patient groups meant that in some cases different individuals represented those groups in 2017.

Following the close of each round of data collection, we reviewed data and queried study participants via email about incomplete, inconsistent or unclear information. We compiled and descriptively analysed final data using Microsoft Excel. We reported 2016 findings in *The 2016 Hep‐CORE Report*, published by ELPA in early 2017 22. The analysis presented in this paper directly compares 2017 findings to findings from parallel 2016 survey questions. Additional File 3 provides country responses to the 2016 and 2017 surveys in comparative tables.

## 3 Results

In both 2016 and 2017, the 25 European liver patient groups that received study invitations all submitted surveys, for a 100% response rate. Box 1 identifies the countries represented by these groups.

Box 1 **Hep‐CORE European study countries**

1

**Table:** 

Austria	Greece	Slovakia
Belgium	Hungary	Slovenia
Bosnia and Herzegovina	Italy	Spain
Bulgaria	Macedonia	Sweden
Croatia	Netherlands	Turkey
Denmark	Poland	Ukraine
Finland	Portugal	United Kingdom
France	Romania	
Germany	Serbia

### 3.1 National coordination

In 2017, patient groups in seven countries (28%) reported that their countries have written national strategies for HBV, and patient groups in 12 countries (48%) reported the same for HCV (Table 1). These findings were similar to 2016 findings, although the survey questions were formulated slightly differently in 2016 and 2017 (Additional Files 1 and 2). The number of patient groups reporting government collaboration with in‐country civil society groups to carry out viral hepatitis prevention and control programmes increased from 13 (52%) in 2016 to 18 (72%) in 2017. In both 2016 and 2017, patient groups reported that nine countries (36%) have disease registers for HBV and 11 (44%) have disease registers for HCV.

**Table 1:** National coordination, monitoring, prevention, screening and treatment policies reported for hepatitis B and hepatitis C in study countries (N=25)

	2016				2017			
	Yes		No	Do not know	Yes		No	Do not know
**National coordination**								
Written national HBV strategy	8 (32%)		17 (68%)	0	7 (28%)		18 (72%)	0
Written national HCV strategy	11 (44%)		14 (56%)	0	12 (48%)		13 (52%)	0
Government collaborates with in‐country civil society groups to plan and carry out its viral hepatitis programme^a^	13 (52%)		9 (36%)	3 (12%)	18^b^ (75%)		5^b^ (21%)	1^b^ (4%)
Government or government‐ related institution has national HBV disease register	9 (36%)		16 (64%)	0	9 (36%)		15 (60%)	1 (4%)
Government or government‐ related institution has national HCV disease register	11 (44%)		14 (56%)	0	11 (44%)		14 (56%)	0
**Prevention**								
Harm reduction services available: Needle and syringe programmes (all parts of country, some parts of country)	All: 10 (40%)	Some: 10 (40%)	4 (16%)	1 (4%)	All: 8 (32%)	Some: 12 (48%)	3 (12%)	2 (8%)
Harm reduction services available: Opioid substitution therapy (all parts of country, some parts of country)	All: 22 (88%)	Some: 1 (4%)	0	2 (8%)	All: 20 (80%)	Some: 4 (16%)	0	1 (4%)
Harm reduction services available: Drug consumption rooms (all parts of country, some parts of country)	All: 2 (8%)	Some: 2 (8%)	17 (68%)	4 (16%)	All: 1 (4%)	Some: 5 (20%)	16 (64%)	3 (12%)
**Screening**								
Risk assessment for HBV/HCV included in routine medical check‐ups	5 (20%)		20 (80%)	0	6 (24%)		18 (72%)	1 (4%)
Liver enzyme testing included in routine medical check‐ups	17 (68%)		8 (32%)	0	14 (56%)		10 (40%)	1 (4%)
**Treatment**								
HBV treatment provided in prisons	18^c^ (75%)		5^c^ (21%)	1^c^ (4%)	19 (76%)		5 (20%)	1 (4%)
HCV patients have option to be treated in non‐hospital settings^d^	5 (20%)		20 (80%)	0	5 (20%)		20 (80%)	0

HBV, hepatitis B virus; HCV, hepatitis C virus.

a Survey respondents were advised that the following are not considered in‐country civil society groups: United Nations agencies, international non‐governmental organizations, government ministries, university programmes and military programmes.

b 2017 responses to this question total 24 instead of 25 because there was one non‐response.

c 2016 responses to this question total 24 instead of 25 because there was one non‐response.

d Settings that are not within either inpatient or outpatient hospital facilities.

### 3.2 Prevention

While patient groups in most study countries reported opioid substitution therapy (OST) to be available in all parts of the country in both 2016 and 2017, far fewer reported needle and syringe programmes (NSPs) to be available in all parts of the country – 10 (40%) in 2016 and 8 (32%) in 2017 (Table 1). Drug consumption rooms were reported to be available in either all or some parts of the country in four countries (16%) in 2016 and six countries (24%) in 2017.

### 3.3 Testing

HBV/HCV risk assessment was reported to be included in routine medical check‐ups in six countries (24%) in 2017, and liver enzyme testing in 14 countries (56%) (Table 1).

**Figure 1:**
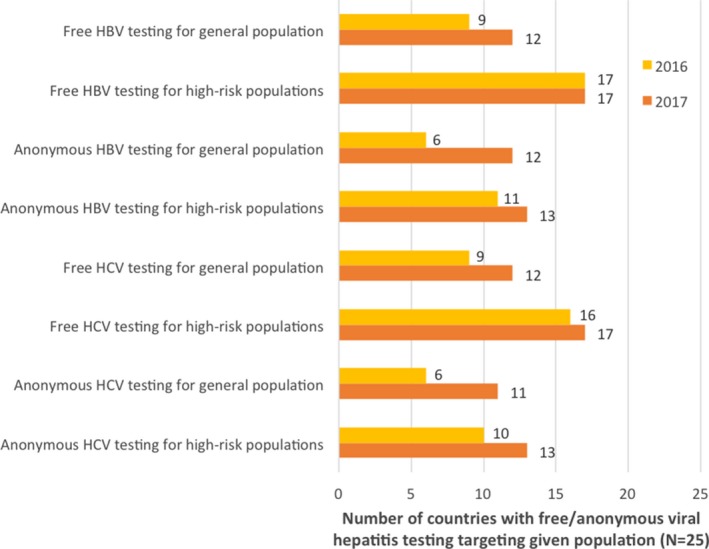
Reported availability of free and anonymous viral hepatitis testing services in study countries (N=25)

Patient groups were asked to indicate whether free and anonymous HBV and HCV testing services targeting the general population and high‐risk populations are available in their countries (Figure 1). From 2016 to 2017, large increases occurred in the number of countries reported to have anonymous HBV and HCV testing for the general population, with anonymous HBV testing up from six to twelve countries and anonymous HCV testing up from six to eleven countries. There also were large increases for free HBV and HCV testing for the general population, and for anonymous HCV testing for high‐risk populations.

Eighteen countries (72%) reported HBV testing availability outside of hospitals in 2017, with the same number of countries reporting HCV testing/screening availability outside of hospitals in that year though with slight variation for HBV and HCV (data not shown). Types of testing sites included general practitioner clinics (HBV, 11 countries; HCV, 10 countries), OST clinics (HBV, 9 countries; HCV, 10 countries) and NSPs (HBV, 5 countries; HCV 7 countries). In 2016, study participants were asked about non‐hospital‐based HBV and HCV testing and screening in a different way, with separate survey questions addressing the general population and high‐risk populations. Eleven countries (44%) in 2016 were reported to have HBV testing sites outside of hospitals for the general population, and 16 (64%), for high‐risk populations. Regarding HCV testing, the reported numbers of countries with non‐hospital‐based sites in 2016 were 13 for the general population (52%) and 16 for high‐risk populations (64%).

### 3.4 Treatment

In 2017, patient groups in 19 countries (76%) reported that HBV treatment is provided in prisons, up from 18 countries (72%) in 2016 (Table 1). In both 2016 and 2017, patient groups in five countries (20%) reported that HCV treatment is available in non‐hospital settings. These included general practitioner clinics (three countries in both 2016 and 2017), liver specialist clinics (one country in 2016 and two countries in 2017), and addiction/OST clinics (three countries in 2016 and two countries in 2017) (data not shown).

**Figure 2:**
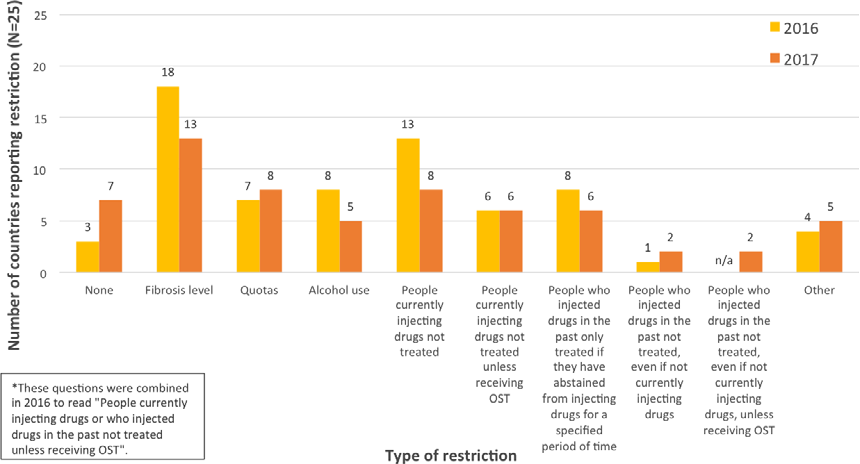
Reported restrictions on access to direct‐acting antivirals for the treatment of hepatitis C in study countries (N=25)

In both 2016 and 2017, survey respondents were asked to choose one or more answers to the question, “In practice, what restrictions are there on access to direct‐acting antivirals for the treatment of HCV infection in your country?” (Figure 2). From 2016 to 2017 the reported number of countries with no restrictions increased from 3 (12%) to 7 (28%). Countries with a reported fibrosis level restriction dropped from 18 (72%) to 13 (52%), and countries reported to refuse treatment to people who are currently injecting drugs dropped from 13 (52%) to 8 (32%).

## 4 Discussion

The Hep‐CORE study was conducted in 2016 and 2017 against the backdrop of rapid changes in the field of viral hepatitis. It found that, from the perspective of liver patient groups, many European countries have shortcomings across the spectrum of elements that constitute a comprehensive policy response to viral hepatitis, including national coordination, public awareness, disease surveillance, prevention, testing and treatment. At the time of 2017 data collection, only 28% and 48% of the 25 European study countries were reported to have national HBV and HCV strategies respectively. National disease registers were reported to be lacking in many countries as well. Some key prevention, testing and treatment recommendations did not appear to be widely reflected in national viral hepatitis control efforts, suggesting that governments may be missing important opportunities to limit disease transmission, disease progression and mortality.

Despite the availability of many simple operational interventions to improve the continuum of care for people with chronic HBV and HCV, relatively few of these people are offered treatment 23, 24. At the same time, there are problematic gaps in disease prevention efforts 23. The Hep‐CORE study provides a patient group perspective on policy barriers that may be undermining progress against HBV and HCV in the European Region, while at the same time offering a model for how patient groups might contribute to policy monitoring in other regions. Findings indicate that few study countries are sufficiently attuned to the importance of addressing the HBV and HCV prevention and treatment needs of the key populations most affected by these diseases in Europe, such as people who inject drugs (PWID). NSPs can contribute substantially to reducing transmission of blood‐borne viruses among PWID 25, 26, yet less than one‐third of study countries were reported to have NSPs available in all parts of their countries in 2017. The presence of injecting drug use‐related restrictions on access to DAAs for the treatment of HCV in several study countries is also a matter of concern, and other research has similarly documented such restrictions 27. European and global experts concur that injecting drug use does not constitute a valid reason for withholding treatment 28, 29, 30 – indeed, global HCV elimination strategies depend on the reductions in transmission that are expected to occur when large numbers of chronically HCV‐infected people who currently inject drugs are cured with DAAs 31.

Hep‐CORE 2016 and 2017 study findings differ regarding the number of countries reported to have components of the recommended HBV and HCV policy infrastructure in place. We hypothesise that some small changes from 2016 to 2017 may be attributable to how respondents interpreted questions differently from one year to the next, or in some cases to changes in how questions were formulated in the study instrument. It is also possible that respondents became more knowledgeable and thus provided more accurate information about some issues in the second round of reporting. We are therefore disinclined to interpret small changes from 2016 to 2017 as trends. The magnitude of some changes, however, is notable. The number of respondents indicating that their governments collaborated with civil society on viral hepatitis control increased considerably from 2016 to 2017, as did the number of respondents reporting the availability of free and anonymous HBV and HCV testing for the general population. Also, the number of study countries reported to have no restrictions on access to DAAs for HCV treatment increased from three to seven, with fewer countries reported to restrict access according to fibrosis or drug injecting status. These changes might partially reflect efforts to encourage European governments to align their responses to viral hepatitis with global and regional guidance. Patient groups’ research inquiries for the 2016 survey and ELPA's extensive dissemination of study findings may have influenced governments as well.

Researchers have proposed that the global HIV policy monitoring process led by UNAIDS can serve as a useful model for other monitoring initiatives, such as those that will be needed to track countries’ efforts to achieve the Sustainable Development Goals 32. The UNAIDS reporting framework repeats some of the same questions in surveys that are completed separately by government and civil society representatives. This makes it possible to examine points of disagreement. Interestingly, an assessment of differences between government reporting and civil society reporting in the aforementioned NCPI found that civil society stakeholders generally characterize national HIV responses less favourably than governments do 33. The discrepancies are allowed to stand as part of the evidence presented in the final country reports 20. This point may be instructive for the viral hepatitis field, where civil society stakeholders have challenged some governments’ accounts of their countries’ policy and programmatic responses to viral hepatitis 16, 17. Researchers have documented increased collaboration between governments and civil society stakeholders on NCPI reporting over time, and have concluded that this approach has strengthened the overall response to HIV 32, 33.

In the field of viral hepatitis, WHO has proposed a monitoring framework for countries to track progress towards the targets in the WHO global strategy 34. The 10 core indicators recommended by WHO ask about disease incidence, prevalence and mortality, as well as a number of key interventions. The emphasis is thus on outputs rather than on indicators of a strong policy environment. It is not known how many countries have adopted the WHO monitoring framework since it was published in April 2016, or when WHO will establish mechanisms for countries to publicly report their monitoring findings. We must consider how such monitoring should be expanded to address policy issues more comprehensively, and how future monitoring processes should be structured, with consideration given to the role of civil society stakeholders such as the patient groups that participated in the Hep‐CORE study. Furthermore, we are not aware of similar data being collected in other regions of the world; conducting such an exercise in other regions would provide the basis for comparative analyses.

Arguably, the central limitation of this study is that patient groups were the only participants. Patient groups, especially those that lack established communication channels with government officials or viral hepatitis experts, may not always be the most well‐placed to report accurately on national policies. However, patients have a distinctly different perspective from other stakeholders, arising from unique motivations. This may enable them to report on policy shortcomings that other observers have failed to identify. In designing a study that gathered policy information only from ELPA patient groups, the research team was both seeking to make optimal use of limited resources and also to emphasize the importance of patient engagement in the viral hepatitis policy discourse. It is hoped that government representatives in all Hep‐CORE study countries will review the information reported for their countries (Additional File 3) and will share their perspectives on this information with the reporting patient groups. Ultimately, governments and other viral hepatitis stakeholders in Europe and elsewhere should have the goal of establishing an ongoing viral hepatitis policy monitoring process that incorporates the expertise of all stakeholders.

Additional study limitations should be noted. The questionnaire was exclusively in English, which may have led to the misinterpretation of questions, despite the study team inviting respondents to ask for clarification on any survey questions that they did not understand. Only one patient group, or in the case of the United Kingdom a coalition of two groups, served as a respondent from each country; thus, the information provided might not reflect the perspectives of other patient groups in the study countries. Because the study focused on patient group reporting, survey answers were not checked against other sources of information, and it is not possible to know whether the information reported is accurate. Some respondents may have been hampered in their reporting efforts by inadequate government communication about existing policies.

## 5 Conclusions

Although planning for the Hep‐CORE study commenced before the official launch of the WHO global health sector strategy on viral hepatitis, it anticipated many of the strategy's key points. As such, Hep‐CORE offers unique insights into the readiness of study countries to pursue hepatitis elimination targets. The finding that warrants the most urgent attention is the reported absence of written national HBV and HCV strategies in many European countries. Other key policy barriers impeding viral hepatitis elimination efforts include widespread restrictions to treatment, limited availability of free and anonymous testing, and insufficient access to testing, prevention and treatment in non‐hospital settings. As Hep‐CORE is driven by patient engagement, it has the potential to foster greater interaction and cooperation between governments and patient groups. Future policy monitoring in the Hep‐CORE countries and elsewhere should incorporate the perspectives of additional stakeholder groups including governments and medical professionals in order to ensure the most reliable reporting, while also reflecting points of disagreement among reporting parties. Frequent rounds of policy monitoring are needed in the light of how rapidly the public health response to viral hepatitis is evolving in Europe and elsewhere.

## Competing interests

The authors declare that they have no competing interests.

## Authors’ contributions

JVL was the Hep‐CORE study′s principal investigator. JVL, SRS and KSH drafted the article with input from the co‐authors. KSH also served as the Hep‐CORE study coordinator and SRS as the data manager. MH, GH, MM, MJR, JT, KS and TR were part of the Hep‐CORE study group and contributed to the development of the original study instrument as well as providing input on the article. All authors reviewed the full draft of the article and approved the final version for submission.

## Funding

The Hep‐CORE study was financed by the European Liver Patients’ Association with support from unrestricted grants by AbbVie Inc., Gilead Sciences Inc., and MSD.

## Supporting information


**Additional File 1.** Hep‐CORE 2016 survey: monitoring the implementation of hepatitis B and C policy recommendations in Europe.

**Additional File 2.** Hep‐CORE 2017 survey.

**Additional File 3**. Detailed comparative data on national coordination, monitoring, prevention, screening and treatment for Hep‐CORE 2016 and 2017 (N = 25 countries).

## References

[1] Stanaway JD , Flaxman AD , Naghavi M , Fitzmaurice C , Vos T , Abubakar I , et al. The global burden of viral hepatitis from 1990 to 2013: findings from the Global Burden of Disease Study 2013. Lancet. 2016;388(10049):1081–8. 27394647 10.1016/S0140-6736(16)30579-7 PMC5100695

[2] World Health Organization . Global health sector strategy on viral hepatitis, 2016–2021: towards ending viral hepatitis [Internet]. 2016 [cited 9 Jan 2018]. Available from: http://apps.who.int/iris/bitstream/10665/246177/1/WHO-HIV-2016.06-eng.pdf?ua=1.

[3] GBD 2015 Mortality and Causes of Death Collaborators . Global, regional, and national life expectancy, all‐cause mortality, and cause‐specific mortality for 249 causes of death, 1980‐2015: a systematic analysis for the Global Burden of Disease Study 2015. Lancet. 2016;388(10053):1459–544. 27733281 10.1016/S0140-6736(16)31012-1 PMC5388903

[4] Perz JF , Armstrong GL , Farrington LA , Hutin YJ , Bell BP . The contributions of hepatitis B virus and hepatitis C virus infections to cirrhosis and primary liver cancer worldwide. J Hepatol. 2006;45(4):529–38. 16879891 10.1016/j.jhep.2006.05.013

[5] Global Burden of Disease Cancer Collaboration , Fitzmaurice C , Dicker D , Pain A , Hamavid H , Moradi‐Lakeh M , MacIntyre MF , et al. The Global Burden of Cancer 2013. JAMA Oncol. 2015;1(4):505–27. 26181261 10.1001/jamaoncol.2015.0735 PMC4500822

[6] Schweitzer A , Horn J , Mikolajczyk RT , Krause G , Ott JJ . Estimations of worldwide prevalence of chronic hepatitis B virus infection: a systematic review of data published between 1965 and 2013. Lancet. 2015;386(10003):1546–55. 26231459 10.1016/S0140-6736(15)61412-X

[7] Hope VD , Eramova I , Capurro D , Donoghoe MC . Prevalence and estimation of hepatitis B and C infections in the WHO European Region: a review of data focusing on the countries outside the European Union and the European Free Trade Association. Epidemiol Infect. 2014;142(02):270–86. 23714072 10.1017/S0950268813000940 PMC3891474

[8] World Health Organization Regional Office for Europe . Action plan for the health sector response to viral hepatitis in the WHO European Region – DRAFT [Internet]. 2016 [cited 9 Jan 2018]. Available from: http://www.euro.who.int/__data/assets/pdf_file/0017/318320/European-action-plan-HS-viral-hepatitis.pdf?ua=1

[9] Chan DPC , Sun H-Y , Wong HTH , Lee S-S , Hung C-C . Sexually acquired hepatitis C virus infection: a review. Int J Infect Dis. 2016;49:47–58. 27270138 10.1016/j.ijid.2016.05.030

[10] Lanini S , Easterbrook PJ , Zumla A , Ippolito G . Hepatitis C: global epidemiology and strategies for control. Clin Microbiol Infect. 2016;22(10):833–8. 27521803 10.1016/j.cmi.2016.07.035

[11] Lazarus JV , Safreed-Harmon K , Maticic M . HMAP, World Hepatitis Day and the bigger health systems picture. Hepatol Med Pol. 2016;2:4.

[12] The Lancet . Eliminating viral hepatitis: time to match visions with action. Lancet. 2017;11(390):2121. 10.1016/S0140-6736(17)32856-8 29143741

[13] Sixty-seventh World Health Assembly . WHA67.6 Hepatitis. [Internet]. 2014 [cited 9 Jan 2018]. Available from: http://apps.who.int/gb/ebwha/pdf_files/WHA67/A67_R6-en.pdf

[14] European Liver Patients’ Association . Hepatitis B and C: an action plan for saving lives in Europe. 2015.

[15] United Nations . Transforming our world: the 2030 Agenda for Sustainable Development. New York: United Nations ; 2015 [cited 9 Jan 2018]. Available from: https://sustainabledevelopment.un.org/post2015/transformingourworld

[16] World Health Organization . Global policy report on the prevention and control of viral hepatitis in WHO Member States. 2013 [cited 9 Jan 2018]. Available from: http://apps.who.int/iris/bitstream/10665/85397/1/9789241564632_eng.pdf

[17] World Hepatitis Alliance . Global community hepatitis policy report. 2014 [cited 9 Jan 2018]. Available from: http://www.worldhepatitisalliance.org/sites/default/files/resources/documents/Community policy report.pdf

[18] Saxena S , Lora A , van Ommeren M , Barrett T , Morris J , Saraceno B . WHO's Assessment Instrument for Mental Health Systems: collecting essential information for policy and service delivery. Psychiatr Serv. 2007;58(6):816–21. 17535942 10.1176/ps.2007.58.6.816

[19] Swinburn B , Vandevijvere S , Kraak V , Sacks G , Snowdon W , Hawkes C , et al. Monitoring and benchmarking government policies and actions to improve the healthiness of food environments: a proposed Government Healthy Food Environment Policy Index. Obes Rev. 2013;14: Suppl 1:24–37. 24074208 10.1111/obr.12073

[20] UNAIDS . Global AIDS monitoring 2017: indicators for monitoring the 2016 United Nations Political Declaration on HIV and AIDS. 2016 [cited 9 Jan 2018]. Available from: http://www.unaids.org/sites/default/files/media_asset/2017-Global-AIDS-Monitoring_en.pdf

[21] Harris PA , Taylor R , Thielke R , Payne J , Gonzalez N , Conde JG . Research electronic data capture (REDCap) ‐ A metadata‐driven methodology and workflow process for providing translational research informatics support. J Biomed Inform. 2009;42(2):377–81. 18929686 10.1016/j.jbi.2008.08.010 PMC2700030

[22] European Liver Patients’ Association . The 2016 Hep-CORE report: monitoring the implementation of hepatitis B and C policy recommendations in Europe. 2017 [cited 9 Jan 2018]. Available from: https://www.elpa-info.org/project/hep-core-study

[23] World Health Organization . Global hepatitis report 2017 [cited 9 Jan 2018]. Available from: http://apps.who.int/iris/bitstream/10665/255016/1/9789241565455-eng.pdf?ua=1

[24] Zhou K , Fitzpatrick T , Walsh N , Kim JY , Chou R , Lackey M , et al. Interventions to optimise the care continuum for chronic viral hepatitis: a systematic review and meta‐analyses. Lancet Infect Dis. 2016;16(12):1409–22. 27615026 10.1016/S1473-3099(16)30208-0

[25] Nosyk B , Zang S , Min JE , Krebs E , Lima VD , Milloy MJ , et al. The relative impacts of ART and harm reduction on HIV incidence in British Columbia. Paper presented at: Conference on Retroviruses and Opportunistic Infections; 2017 Feb 13-16; Seattle, WA.

[26] WHO . UNODC, UNAIDS technical guide for countries to set targets for universal access to HIV prevention, treatment and care for injecting drug users – 2012 revision. Geneva: World Health Organization; 2012.

[27] Marshall AD , Cunningham EB , Nielsen S , Aghemo A , Alho H , Backmund M , et al. Restrictions for reimbursement of interferon‐free direct‐acting antiviral drugs for HCV infection in Europe. Lancet Gastroenterol Hepatol. 2017;66: S95–332. Epub 2017 Oct 3. 10.1016/S2468-1253(17)30284-4 28986139

[28] European Association for the Study of the Liver . EASL recommendations on treatment of hepatitis C 2016. J Hepatol. 2017;66(1):153–94. 27667367 10.1016/j.jhep.2016.09.001

[29] EASL Recommendations for Treatment of Hepatitis C 2016 Panel. Electronic address: easloffice@easloffice.eu Reply to: “Contradictory advice for people who inject drugs in the 2016 EASL Recommendations on Treatment of Hepatitis C”. J Hepatol. 2017;66(5):1103. 28167321 10.1016/j.jhep.2017.01.021

[30] World Health Organization . Guidelines for the screening, care and treatment of persons with hepatitis C infection: updated version. 2016 [cited 31 Jan 2017]. Available from: http://apps.who.int/iris/bitstream/10665/205035/1/9789241549615_eng.pdf?ua=1 27227200

[31] Grebely J , Dore GJ , Morin S , Rockstroh JK , Klein MB . Elimination of HCV as a public health concern among people who inject drugs by 2030 ‐ What will it take to get there? J Int AIDS Soc. 2017;20(1):22146. 28782335 10.7448/IAS.20.1.22146 PMC5577699

[32] Torres MA , Gruskin S , Buse K , Erkkola T , Bendaud V , Alfvén T . Monitoring HIV‐related laws and policies: lessons for AIDS and global health in agenda 2030. AIDS Behav. 2017;21:51–61. 10.1007/s10461-016-1621-5 28084561

[33] Peersman G , Ferguson L , Torres MA , Smith S , Gruskin S . Increasing civil society participation in the national HIV response: the role of UNGASS reporting. J Acquir Immune Defic Syndr. 2009;52 Suppl 2:S97–103. 19901632 10.1097/QAI.0b013e3181baee06

[34] World Health Organization . Monitoring and evaluation for viral hepatitis B and C: recommended indicators and framework. 2016 [cited 9 Jan 2018]. Available from: http://apps.who.int/iris/bitstream/10665/204790/1/9789241510288_eng.pdf?ua=1

