# Supplementary material for: Hep‐CORE: a cross‐sectional study of the viral hepatitis policy environment reported by patient groups in 25 European countries in 2016 and 2017
Source: J Int AIDS Soc. 2018 Apr 10;21(Suppl Suppl 2):e25052. doi: 10.1002/jia2.25052 (PMC5978657; doi:10.1002/jia2.25052)
Supplement: Supplementary file 1 — Additional File 1. Hep‐CORE 2016 survey: monitoring the implementation of hepatitis B and C policy recommendations in Europe. [file JIA2-21-e25052-s001.docx]

**Hep-CORE 2016 survey: monitoring the implementation of hepatitis B and C policy recommendations in Europe**

Thank you for participating in the 2016 Hep-CORE study.

Please note:

A PDF of the survey is available for reference. The actual survey must be completed online. To download the PDF, use the link below.

To move from one section of the survey to another, please use the arrow buttons at the bottom of each section. Do not use the back and forward buttons in your browser.

To save your work and continue later, use the Save & Return Later button.

Please fully answer all questions before clicking on the Submit button on the last page.

If you do not understand a survey question or would like help finding the information that is being requested, please contact Kelly Safreed-Harmon at [kelly@safreed-harmon.com.](mailto:kelly@safreed-harmon.com)

If you have any other questions about the Hep-CORE study, please contact Principal Investigator Jeffrey V. Lazarus of the University of Copenhagen at [jeffrey.lazarus@regionh.dk.](mailto:jeffrey.lazarus@regionh.dk)

**Contact Information**

First name:

Last name:

Organisation:

Position:

Street address:

City:

Postal code:

Country:

Phone number:

E-mail address:

**SECTION 1. OVERALL NATIONAL RESPONSE**

- 1. Written national HBV and/or HCV strategy

Does your country have a written national HBV and/or HCV strategy?

Which best describes the content of the strategy?

Is there an action plan stating how the strategy will be implemented?

Is the strategy exclusively for viral hepatitis, or does it integrate viral hepatitis with other diseases?

Please provide an internet URL (a link) to the document or provide an e-mail address for a contact person who has the document

Additional comments:

Sources for answers:

- Yes
- No
- It includes both HBV and HCV
- It includes HBV but not HCV
- It includes HCV but not HBV
- Yes
- No
- Do not know
- It is exclusive for viral hepatitis
- It integrates viral hepatitis with other diseases
- Do not know

1.2 National clinical guidelines for the diagnosis and treatment of HBV

Does your country have national clinical guidelines for the diagnosis & treatment of HBV?

What is the source of the guidelines?

Other source of the guidelines, please specify:

Additional comments:

Sources for answers:

- Yes
- No
- Do not know
- Guidelines by European Association for the Study of the Liver (EASL) or other international clinical association are adopted as national guidelines
- Guidelines by World Health Organization (WHO) are adopted as national guidelines
- National government develops its own national guidelines
- National medical society develops its own national guidelines
- Other (please specify below
- Do not know

1.3 National clinical guidelines for the diagnosis and treatment of HCV

Does your country have national clinical guidelines for the diagnosis & treatment of HBV?

What is the source of the guidelines?

Other source of the guidelines, please specify:

Additional comments:

Sources for answers:

- Yes
- No
- Do not know
- Guidelines by European Association for the Study of the Liver (EASL) or other international clinical association are adopted as national guidelines
- Guidelines by World Health Organization (WHO) are adopted as national guidelines
- National government develops its own national guidelines
- National medical society develops its own national guidelines
- Other (please specify below
- Do not know

1.4 Multidisciplinary/technical advisory/Ministry of Health working group for viral hepatitis

Does your national government have a multidisciplinary/technical advisory/ Ministry of Health working group for viral hepatitis?

How often does it meet?

Additional comments:

Sources for answers:

- Yes
- No
- Do not know
- Less than once per year
- Once per year or more
- Has not yet held first meeting
- Do not know

1.5 National laws that protect people against discrimination based on their HBV/HCV status

In your country are there any national laws that protect people against discrimination based on their HBV/HCV status?

Please identify law by name (1):

Please identify law by name (2):

Please identify law by name (3):

- Yes
- No
- Do not know

Additional comments:

Sources for answers:

**SECTION 2. PUBLIC AWARENESS AND ENGAGEMENT**

2.1 Events or awareness campaigns for World Hepatitis Day 2015

Did your government stage events or awareness campaigns for World Hepatitis Day 2015?

At what level? Please choose all answers that apply.

Were civil society groups involved? Click to see a definition of “civil society groups”

Additional comments:

Sources for answers:

- Yes
- No
- Do not know
- National government
- Subnational governments (e.g., province, region), with all participating
- Subnational governments (e.g., province, region), with at least 50% of governments (≥ 50%) participating
- Subnational governments (e.g., province, region), with less than 50% of governments (<50%) participating
- Do not know
- Yes
- No
- Do not know

2.2 Events or awareness campaigns for World Hepatitis Day 2016

Is your government planning to stage events or awareness campaigns for World Hepatitis Day 2016?

At what level? Please choose all answers that apply.

- Yes
- No
- Do not know
- National government
- Subnational governments (e.g., province, region), with all participating
- Subnational governments (e.g., province, region), with at least 50% of governments (≥ 50%) participating
- Subnational governments (e.g., province, region), with less than 50% of governments (<50%) participating
- Do not know

Were civil society groups involved? Click to see a definition of “civil society groups”

Additional comments:

Sources for answers:

- Yes
- No
- Do not know

2.3 Viral hepatitis awareness campaigns since January 2015, other than World Hepatitis Day

Has your government funded – directly or via an NGO – any viral hepatitis awareness campaigns since January 2015, other than World Hepatitis Day? Click to see a definition of “NGO”

At what level? Please choose all answers that apply.

What were the campaign channels? Please choose all answers that apply.

Other campaign channel, please specify (1):

Other campaign channel, please specify (2):

Other campaign channel, please specify (3):

Who were the target groups? Please choose all answers that apply

Other target population, please specify (1):

- Yes
- No
- Do not know
- National government
- Subnational governments (e.g., province, region), with all participating
- Subnational governments (e.g., province, region), with at least 50% of governments (≥ 50%) participating
- Subnational governments (e.g., province, region), with less than 50% of governments (<50%) participating
- Do not know
- Mass media
- Social Media
- Public events
- Other (please specify below)
- Do not know
- General population
- People who inject drugs
- Men who have sex with men
- Transgender people Sex workers Prisoners
- Healthcare workers
- Migrants
- People living with HIV
- Other (please specify below)
- Do not know

Other target population, please specify (2):

Other target population, please specify (3):

What were the primary topics or messages? Please choose all answers that apply.

Other primary topic or message, please specify (1):

Other primary topic or message, please specify (2):

Other primary topic or message, please specify (3):

Additional comments:

Sources for answers:

- General information about viral hepatitis
- Importance of HBV vaccination
- Importance of knowing one's HBV and/or HCV status
- Importance of safer sex
- Harm reduction for people who inject drugs Viral hepatitis prevention in healthcare settings Other (please specify below)
- Do not know

2.4 Government collaboration with any in-country civil society groups

Does your government collaborate with any in-country civil society groups (such as patient groups, community groups or local or national NGOs) within your country to plan and carry out its viral hepatitis prevention and control programme? (The following are not considered in-country civil society groups: United Nations agencies, international NGOs, government ministries, university programmes, military programmes.) Click to see definitions of "civil society groups" and "NGOs".

Please name group (1):

Please name group (2):

Please name group (3):

Please name group (4):

Please name group (5):

Please name group (6):

Additional comments:

Sources for answers:

- Yes
- No
- Do not know

2.5 Viral hepatitis awareness activities targeting healthcare workers

Since January 2015, has any government agency carried out viral hepatitis awareness activities targeting healthcare workers?

At what level? Please choose all answers that apply.

Please briefly describe activities

Additional comments:

Sources for answers:

- Yes
- No
- Do not know
- National government
- Subnational governments (e.g., province, region), with all participating
- Subnational governments (e.g., province, region), with at least 50% of governments (≥ 50%) participating
- Subnational governments (e.g., province, region), with less than 50% of governments (<50%) participating
- Do not know

**SECTION 3. MONITORING AND DATA COLLECTION**

3.1 National government employs a "cascade of care" approach to monitor the numbers and proportions of people who progress through each stage of the HBV and HCV care cascades.

Does your national government employ a "cascade of care" approach to monitor the numbers and proportions of people who progress through each stage of the HBV and HCV care cascades? (Stages such as testing, diagnosis, linkage to care, assessment, treatment and sustained viral response.) Click to see a definition of "cascade of care".

Additional comments:

Sources for answers:

- Yes
- No
- Do not know

3.2 National disease register for HBV infection

Does your government or any government-related institution have a national disease register for HBV infection? Click to see a definition of "disease register"

- Yes
- No
- Do not know

Are data collected from mandatory notification of every case of HBV infection?

Are subnational level (e.g., province, region) data available?

Additional comments:

Sources for answers:

- Yes
- No
- Do not know
- Yes
- No
- Do not know

3.3 National disease register for HCV infection

Does your government or any government-related institution have a national disease register for HCV infection? Click to see a definition of "disease register"

Are data collected from mandatory notification of every case of HBV infection?

Are subnational level (e.g., province, region) data available?

Additional comments:

Sources for answers:

- Yes
- No
- Do not know
- Yes
- No
- Do not know
- Yes
- No
- Do not know

3.4 National disease register for HCC infection

Does your government or any government-related institution have a national disease register for HCV infection? Click to see a definition of "disease register"

Are data collected from mandatory notification of every case of HBV infection?

Are subnational level (e.g., province, region) data available?

Additional comments:

Sources for answers:

- Yes
- No
- Do not know
- Yes
- No
- Do not know
- Yes
- No
- Do not know

**SECTION 4. PREVENTION**

4.1 National policy to address prevention of HBV/HCV infection in healthcare settings

Is there a national policy that addresses prevention of HBV/HCV infection in healthcare settings?

What topics does the policy address? Please choose all answers that apply.

Other topic, please specify (1)

Other topic, please specify (2)

Other topic, please specify (3)

Additional comments:

Sources for answers:

- Yes
- No
- Do not know
- HBV vaccination for healthcare workers
- National HBV/HCV prevention and control regulations/protocols
- Universal blood and body fluid precautions (use of protective barriers such as gloves, masks, gowns and eyewear)
- Safe injections (e.g., use of single-use or auto-disable syringes)
- Post-exposure management and prophylaxis for healthcare workers - click to see a definition of "prophylaxis"
- Safe medical waste management
- Other (please specify below)

4.2 HBV prevention addressed in different populations in your country

How is HBV prevention (other than vaccination) addressed in the following populations in your country?

In the following table, please mark all boxes that apply. If HBV prevention is not addressed in a population in the way described, then leave the box unmarked.

|  | HBV prevention for this population is addressed in national policy | HBV prevention for this population is addressed in subnational HBV policies in all provinces/regions | HBV prevention for this population is addressed in national HBV strategy | HBV prevention for this population is addressed in subnational HBV strategies in all provinces/regions | HBV prevention for this population is addressed in national clinical guidelines |
| --- | --- | --- | --- | --- | --- |
| People who inject drugs |  |  |  |  |  |
| Men who have sex with men |  |  |  |  |  |
| Transgender people |  |  |  |  |  |
| Sex workers |  |  |  |  |  |
| Prisoners |  |  |  |  |  |
| Migrants |  |  |  |  |  |
| People living with HIV |  |  |  |  |  |
| Other (Please specify below) |  |  |  |  |  |

Other population, please specify:

Additional comments:

Sources for answers:

4.3 HCV prevention addressed in different populations in your country

How is HCV prevention (other than vaccination) addressed in the following populations in your country?

In the following table, please mark all boxes that apply. If HCV prevention is not addressed in a population in the way described, then leave the box unmarked.

|  | HCV prevention for this population is addressed in national policy | HCV prevention for this population is addressed in subnational HCV policies in all provinces/regions | HCV prevention for this population is addressed in national HCV strategy | HCV prevention for this population is addressed in subnational HCV strategies in all provinces/regions | HCV prevention for this population is addressed in national clinical guidelines |
| --- | --- | --- | --- | --- | --- |
| People who inject drugs |  |  |  |  |  |
| Men who have sex with men |  |  |  |  |  |
| Transgender people |  |  |  |  |  |
| Sex workers |  |  |  |  |  |
| Prisoners |  |  |  |  |  |
| Migrants |  |  |  |  |  |
| People living with HIV |  |  |  |  |  |
| Other (Please specify below) |  |  |  |  |  |

Other population, please specify:

Additional comments:

Sources for answers:

4.4 Screening for HBV and HCV in blood, tissue and organ donations

|  | Screened using only serological tests (HBsAg, anti-HBc | Screened using only NAT test (HBV DNA) | Screened using both serological tests (HbsAg, anti-HBc) and NAT test (HBV DNA) | Not screened | Do not know |
| --- | --- | --- | --- | --- | --- |
| HBV - Blood and blood products |  |  |  |  |  |
| HBV - Tissue and organ donations |  |  |  |  |  |
|  | Screened using only serological test (anti-HCV) | Screened using only NAT test (HCV RNA) | Screened using both serological test (anti-HCV) and NAT test (HCV RNA) | Not screened | Do not know |
| HCV - Blood and blood products |  |  |  |  |  |
| HCV - Tissue and organ donations |  |  |  |  |  |

Additional comments:

Sources for answers:

4.5 Routine HBV vaccination

Which populations are routinely vaccinated for HBV in your country, and how are vaccination costs met?

Please mark all boxes that apply in the following table. (More than one box can be marked per row if vaccination costs are met in more than one way.) If you do not know an answer, please leave the box empty.

|  | Free vaccination | Co-payment required for vaccination | Full out-of-pocket payment required for vaccination |
| --- | --- | --- | --- |
| Everyone |  |  |  |
| Travellers |  |  |  |
| Military personnel |  |  |  |
| Healthcare workers |  |  |  |
| Individuals other than healthcare workers who are at risk for HBV due to occupation (including environmental and sanitary workers) |  |  |  |
| Neonates (infants under 28 days of age) born to HbsAg-positive mothers |  |  |  |
| All neonates (infants under 28 days of age) |  |  |  |
| All infants (children aged < 365 days) |  |  |  |
| People who inject drugs |  |  |  |
| Migrants, including refugees and asylum seekers |  |  |  |
| Prisoners |  |  |  |
| Haemodialysis patients |  |  |  |
| Chronic liver disease patients (e.g., people with chronic HCV, alcoholic liver disease, non-alcoholic fatty liver disease) |  |  |  |
| Sexually transmitted infection (STI) clinic patients |  |  |  |
| People living with HIV |  |  |  |
| People with multiple sexual partners |  |  |  |
| Men who have sex with men |  |  |  |
| Transgender people |  |  |  |
| Contacts of HBV-infected people |  |  |  |
| Sex workers |  |  |  |
| Other (Please specify below) |  |  |  |

Other population, please specify:

Additional comments:

Sources for answers:

4.6 National campaigns promoting safer sex as an HBV/HCV prevention strategy

Since January 2015, has your government or any government-related institution conducted or funded an NGO to conduct any national campaigns promoting safer sex as an HBV/HCV prevention strategy? Click to see a definition of "NGO".

Which populations were targeted? Please choose all answers that apply.

Other population, please specify (1):

Other population, please specify (2):

Other population, please specify (3):

Additional comments:

Sources for answers:

- Yes
- No
- Do not know
- General population
- People who inject drugs
- Transgender people
- Sex workers
- Prisoners
- Migrants
- Adolescents/young adults
- People with sexually transmitted infections (STIs)
- Other (please specify below)
- Do not know

4.7 Harm reduction services available to people who inject drugs

In your country, which of the following harm reduction services are available to people who inject drugs?

|  | Available in all parts of the country | Available only in some parts of the country | Not available | Do not know |
| --- | --- | --- | --- | --- |
| Needle and syringe programmes |  |  |  |  |
| Opioid substitution therapy |  |  |  |  |
| Drug consumption rooms |  |  |  |  |
| Other (Please specify below) |  |  |  |  |

Other harm reduction service, please specify (1):

Other harm reduction service, please specify (2):

Other harm reduction service, please specify (3):

Additional comments:

Sources for answers:

4.8 Harm reduction services available in prisons

In your country, which of the following harm reduction services are available in prisons?

|  | Available in prisons in all parts of the country | Available in prisons in only some parts of the country | Not available in prisons | Do not know |
| --- | --- | --- | --- | --- |
| Needle and syringe programmes |  |  |  |  |
| Opioid substitution therapy |  |  |  |  |
| Other (Please specify below) |  |  |  |  |

Other harm reduction service, please specify:

Additional comments:

Sources for answers:

**SECTION 5. TESTING AND DIAGNOSIS**

5.1 HBV testing/screening sites outside of hospitals for the general population and for high-risk populations

In your country, are there any HBV testing/screening sites outside of hospitals for the general population? (Sites that are not within either inpatient or outpatient hospital facilities.)

- Yes
- No
- Do not know

Please list the types of non-hospital settings where HBV testing is available for the general population:

Type (1):

Type (2):

Type (3):

Type (4):

In your country, are there any HBV testing/screening sites outside of hospitals for high-risk populations? (Sites that are not within either inpatient or outpatient hospital facilities.) Click to see a definition of "high-risk populations".

- Yes
- No
- Do not know

Please list the types of non-hospital settings where HBV testing is available for high-risk populations:

Type (1):

Type (2):

Type (3):

Type (4):

Which high-risk populations? Please choose all answers that apply

- General population
- People who inject drugs
- Men who have sex with men
- Transgender people
- Sex workers
- Prisoners
- Healthcare workers
- Migrants
- People living with HIV
- Other (please specify below)
- Do not know

Other high-risk populations, please specify:

Additional comments:

Sources for answers:

5.2 HCV testing/screening sites outside of hospitals for the general population and for high-risk populations

In your country, are there any HCV testing/screening sites outside of hospitals for the general population? (Sites that are not within either inpatient or outpatient hospital facilities.)

- Yes
- No
- Do not know

Please list the types of non-hospital settings where HCV testing is available for the general population:

Type (1):

Type (2):

Type (3):

Type (4):

In your country, are there any HCV testing/screening sites outside of hospitals for high-risk populations? (Sites that are not within either inpatient or outpatient hospital facilities.) Click to see a definition of "high-risk populations".

- Yes
- No
- Do not know

Please list the types of non-hospital settings where HCV testing is available for high-risk populations:

Type (1):

Type (2):

Type (3):

Type (4):

Which high-risk populations? Please choose all answers that apply

- General population
- People who inject drugs
- Men who have sex with men
- Transgender people
- Sex workers
- Prisoners
- Healthcare workers
- Migrants
- People living with HIV
- Other (please specify below)
- Do not know

Other high-risk populations, please specify:

Additional comments:

Sources for answers:

5.3 Pregnant women routinely screened for HBV and HCV

Are pregnant women in your country routinely screened for HBV?

Are pregnant women in your country routinely screened for HCV?

Additional comments:

Sources for answers:

- Yes
- No
- Do not know
- Yes
- No
- Do not know

5.4 Notification to blood donors if screening of their blood indicates that they have been infected with HBV or HCV

Are blood donors in your country informed if screening of their blood indicates that they have been infected with HBV or HCV?

Are they provided with referrals to medical care?

- Yes
- No
- Do not know
- Yes
- No
- Do not know

Additional comments:

Sources for answers:

5.5 Liver enzyme and/or risk assessment for HBV/HCV in routine medical check-ups

Is liver enzyme testing included in routine medical check-ups in your country?

Is risk assessment for HBV/HCV included in routine medical check-ups in your country?

Additional comments:

Sources for answers:

- Yes
- No
- Do not know
- Yes
- No
- Do not know

5.6 Free and anonymous HBV/HCV testing services targeting high-risk populations

Are there free and anonymous HBV/HCV testing services targeting high-risk populations in your country? Click to see a definition of "high-risk populations".

Please mark all boxes that apply in the following table. If you do not know an answer, please leave the box empty.

|  | There is free HBV testing targeting this population | There is anonymous HBV testing targeting this population | There is free HCV testing targeting this population | There is anonymous HCV testing targeting this population |
| --- | --- | --- | --- | --- |
| General population |  |  |  |  |
| People who inject drugs |  |  |  |  |
| Men who have sex with men |  |  |  |  |
| Transgender people |  |  |  |  |
| Sex workers |  |  |  |  |
| Prisoners |  |  |  |  |
| Migrants |  |  |  |  |
| People living with HIV |  |  |  |  |
| Other (please specify below) |  |  |  |  |

Other populations, please specify:

Additional comments:

Sources for answers:

**SECTION 6. CLINICAL ASSESSMENT**

6.1 Linkage-to-care mechanism

In your country, is there a clear linkage-to-care mechanism so that people who are diagnosed with HBV and HCV are referred directly to a physician who can manage their care? (Either a general practitioner/primary care physician or an appropriate specialist depending on your country's standard practice.) Click to see a definition of "linkage-to-care mechanism".

- Yes
- No
- Do not know

Additional comments:

Sources for answers:

6.2 Monitoring plans in national clinical guidelines for HBV and HCV management

If your country has national clinical guidelines for HBV management, do they include guidelines on how patients should be monitored (e.g., individual patient plans) to prevent the disease from getting worse?

If your country has national clinical guidelines for HCV management, do they include guidelines on how patients should be monitored (e.g., individual patient plans) to prevent the disease from getting worse?

- There are no national clinical guidelines for HBV management
- There are national clinical guidelines for HBV management, and they include guidelines on patient monitoring
- There are national clinical guidelines for HBV management, but they do not include guidelines on patient monitoring
- Do not know
- There are no national clinical guidelines for HCV management
- There are national clinical guidelines for HCV management, and they include guidelines on patient monitoring
- There are national clinical guidelines for HCV management, but they do not include guidelines on patient monitoring
- Do not know

Additional comments:

Sources for answers:

6.2 Monitoring plans in national clinical guidelines for HBV and HCV management

If your country has national clinical guidelines for HBV management, do they include guidelines on how to assess viral hepatitis patients for alcohol use and make referrals

- There are no national clinical guidelines for HBV management
- There are national clinical guidelines for HBV management, and they include

If your country has national clinical guidelines for HBV management, do they include guidelines on how to assess viral hepatitis patients for alcohol use and make referrals

guidelines on assessment for alcohol use and referral to risk reduction and addiction counseling.

- There are national clinical guidelines for HBV management, but they do not include guidelines on assessment for alcohol use and referral to risk reduction and addiction counseling.
- Do not know
- There are no national clinical guidelines for HCV management
- There are national clinical guidelines for HCV management, and they include guidelines on assessment for alcohol use and referral to risk reduction and addiction counseling.
- There are national clinical guidelines for HCV management, but they do not include guidelines on assessment for alcohol use and referral to risk reduction and addiction counseling.
- Do not know

Additional comments:

Sources for answers:

6.4 Average waiting time for liver specialist appointments for patients diagnosed with HBV/HCV

In practice, is the average waiting time for liver specialist appointments longer than six weeks for patients diagnosed with HBV/HCV in your country?

What is the average waiting time, in weeks?

- Yes
- No
- Do not know
- 6 weeks
- 7 weeks
- 8 weeks
- 9 weeks
- 10 weeks
- 11 weeks
- 12 weeks
- 13 weeks
- 14 weeks
- 15 weeks
- 16 weeks
- 17 weeks
- 18 weeks
- 19 weeks
- between 20 and 29 weeks
- between 30 and 39 weeks
- between 40 and 49 weeks
- between 50 and 52 weeks
- More than 52 weeks

Additional comments:

Sources for answers:

**SECTION 7. TREATMENT**

7.1 Availability of drugs for people diagnosed with HBV

Which of the following drugs are available to all patients diagnosed with HBV in your country? Please choose all answers that apply.

- Adefovir
- Emtricitabine
- Entecavir
- Lamivudine
- Pegylated interferon
- Telbivudine
- Tenofovir
- None of the above drugs are available to all patients
- Do not know

Which best describes the cost to patients?

|  | Free treatment | Co-payment required | Out-of-pocket | Other (please specify below |
| --- | --- | --- | --- | --- |
| Adefovir |  |  |  |  |
| Emtricitabine |  |  |  |  |
| Entecavir |  |  |  |  |
| Lamivudine |  |  |  |  |
| Pegylated interferon |  |  |  |  |
| Telbivudine |  |  |  |  |
| Tenofovir |  |  |  |  |

Adefovir - Other, please specify:

Emtricitabine - Other, please specify:

Entecavir - Other, please specify:

Lamivudine - Other, please specify:

Pegylated interferon - Other, please specify:

Telbivudine - Other, please specify:

Tenofovir - Other, please specify:

Additional comments:

Sources for answers:

7.2 Availability of drugs for people diagnosed with HCV

Which of the following drugs are available to all patients diagnosed with HCV in your country? Please choose all answers that apply.

- Daclatasvir
- Dasabuvir
- Ledipasvir/Sofosbuvir
- Ombitasvir/Paritaprevir/Ritonavir
- Sofosbuvir
- None of the above drugs are available to all patients
- Do not know

Which best describes the cost to patients?

|  | Free treatment | Co-payment required | Out-of-pocket | Other (please specify below |
| --- | --- | --- | --- | --- |
| Daclatasvir |  |  |  |  |
| Dasabuvir |  |  |  |  |
| Ledipasvir/Sofosbuvir |  |  |  |  |
| Ombitasvir/Paritaprevir/ Ritonavir |  |  |  |  |
| Sofosbuvir |  |  |  |  |

Daclatasvir - Other, please specify:

Dasabuvir - Other, please specify:

Ledipasvir/Sofosbuvir - Other, please specify:

Ombitasvir/Paritaprevir/ Ritonavir - Other, please specify:

Sofosbuvir- Other, please specify:

Additional comments:

Sources for answers:

7.3 Treatment of HCV patients in non-hospital settings

Do any HCV patients in your country have the option of being treated in non-hospital settings? (Settings that are not within either inpatient or outpatient hospital facilities.)

What type(s) of non-hospital settings?

- Yes
- No
- Do not know
- General practitioner clinics
- Addiction/opioid substitution therapy clinics
- Other (please specify below)

Other type, please specify (1):

Other type, please specify (2):

Other type, please specify (3):

Additional comments:

Sources for answers:

7.4 HBV/HCV treatment in all parts of country

Can HBV treatment be obtained in all parts of your country?

Can HCV treatment be obtained in all parts of your country?

- Yes
- No
- Do not know
- Yes
- No
- Do not know

Additional comments:

Sources for answers:

7.5 HBV and HCV treatment provided in prisons

Is HBV treatment provided in prisons in your country?

What percentage of prisons provide HBV treatment?

Is HCV treatment provided in prisons in your country?

What percentage of prisons provide HCV treatment?

- Yes
- No
- Do not know
- Do not know
- 0-10%
- 10-19%
- 20-29%
- 30-39%
- 40-49%
- 50-59%
- 60-69%
- 70-79%
- 80-89%
- 90-99%
- 100%
- Yes
- No
- Do not know
- Do not know
- 0-10%
- 10-19%
- 20-29%
- 30-39%
- 40-49%
- 50-59%
- 60-69%
- 70-79%
- 80-89%
- 90-99%
- 100%

Additional comments:

Sources for answers:

7.6 Restrictions on access to direct-acting antivirals for the treatment of HCV infection

In practice, what restrictions are there on access to direct-acting antivirals for the treatment of HCV infection in your country? Please choose all answers that apply.

- None
- Fibrosis level: only patients above a certain fibrosis level are eligible for treatment
- Quotas: only a limited number of patients can be treated within a certain time period or a certain geographic area
- Alcohol use: people who currently drink alcohol are not treated
- Injecting drug use: people who injected drugs in the past are not treated, even if they are not currently injecting drugs
- Injecting drug use: people who are currently injecting drugs are not treated
- Injecting drug use: people who injected drugs in the past are only treated if they have abstained from injecting drugs for a specified period of time
- Injecting drug use: people who currently inject drugs or injected drugs in the past are only treated if they are receiving opioid substitution therapy
- Other restrictions (please describe below)
- Do not know

Other restriction, please describe (1):

Other restriction, please describe (2):

Other restriction, please describe (3):

Additional comments:

Sources for answers:

7.7 Licensing to prescribe direct-acting antivirals to HCV patients

Who is licensed to prescribe direct-acting antivirals to HCV patients in your country? Please choose all answers that apply.

- Hepatologists
- Infectious disease physicians
- Gastroenterologists
- Internists
- HIV/AIDS physicians
- General practitioners/primary care physicians
- Other (please specify below)

Other, please specify:

Additional comments:

Sources for answers:

NOTICE

ONLY click on the "Submit" button below if you have completed your work on the survey.

If instead you wish to save your work and continue responding to the survey at a later time, please click on the "Save & Return Later" button.

**– END OF HEP-CORE 2016 SURVEY –**
