# Supplementary material for: Hep‐CORE: a cross‐sectional study of the viral hepatitis policy environment reported by patient groups in 25 European countries in 2016 and 2017
Source: J Int AIDS Soc. 2018 Apr 10;21(Suppl Suppl 2):e25052. doi: 10.1002/jia2.25052 (PMC5978657; doi:10.1002/jia2.25052)
Supplement: Supplementary file 2 — Additional File 2. Hep‐CORE 2017 survey. [file JIA2-21-e25052-s002.pdf]

Hep-CORE 2017 Survey

Thank you for participating in the 2017 Hep-CORE study.

Please note:

A PDF of the survey is available for reference. The actual survey must be completed online. To download the PDF, use the link below.

To move from one page of the survey to another, please use the arrow buttons at the bottom of each section. Do not use the “back” and “forward” buttons in your browser.

To pause the survey, save your work and return to it at any time, click "Save & Return Later" located at the bottom of each page. You must retain the automatically generated Return Code to use to re-enter the survey.

This questionnaire consists of 11 questions and their sub-questions. Though it can be completed in one session, it is recommended to complete the information in multiple sessions in order to have time to reach out to the appropriate experts who can help you find information for some questions.

Please fully answer all questions, even if it takes multiple sessions, before clicking on the “Submit” button on the last page.

To add further details, context, and/or practical information about patient experience for your answers, please utilize the "Additional comments" boxes after each question.

If you do not understand a survey question or would like help finding the information that is being requested, please contact Kelly Safreed-Harmon at kelly@safreed-harmon.com.

If you have any other questions about the Hep-CORE study, please contact Principal Investigator Jeffrey V. Lazarus of the Barcelona Institute for Global Health (ISGlobal) and the University of Copenhagen at jeffrey.lazarus@regionh.dk.

PDF version for reference

[Attachment: "HepCORE_2017_Survey.pdf"]

This PDF of the survey is available for reference only. The survey must be completed online.

Contact Information

All personal and patient group contact information will remain private and all responses to the survey will be reported according to country. Please provide the following information for the person who will be most responsible for completing this survey.

First name: ________________________

Last name: ________________________

Organisation: ________________________

Position: ________________________

Street address: ________________________

Postal code: ________________________

City: ________________________

Country: ________________________

Phone number:
(Include country code, e.g. +45) ________________________

Email address: ________________________

Hep-CORE 2017 Questionnaire

1. Written national strategy and/or action plan

1.1 Does your country have a written national HBV strategy and/or action plan that has been approved or ratified by the government?

◯ Yes
◯ No

Has implementation of the HBV strategy and/or action plan begun?

◯ Yes
◯ No
◯ Do not know

Please provide an internet URL (a link) to the HBV strategy and/or action plan or provide an e-mail address for a contact person who has the document(s):

____

1.2 Does your country have a written national HCV strategy and/or action plan that has been approved or ratified by the government?

◯ Yes
◯ No

Has implementation of the HCV strategy and/or action plan begun?

◯ Yes
◯ No
◯ Do not know

Please provide an internet URL (a link) to the HCV strategy and/or action plan or provide an e-mail address for a contact person who has the document(s):

____

Additional comments:

____

Sources for answers:

____

2. Government collaboration with in-country civil society groups

2.1 Does your government collaborate with any in-country civil society groups (such as patient groups, community groups or local or national NGOs) within your country to plan and carry out its viral hepatitis prevention and control programme?

○ Yes
○ No
○ Do not know

(The following are not considered in-country civil society groups: United Nations agencies, international NGOs, government ministries, university programmes, military programmes.)

Click to see definitions of "civil society groups" and "NGOs".

Please name group (1): ______________________

Please name group (2): ______________________

Please name group (3): ______________________

Please name group (4): ______________________

Please name group (5): ______________________

Please name group (6): ______________________

Additional comments: ______________________

Sources for answers: ______________________

3. National government employs a "cascade of care" approach to HBV and HCV monitoring

3.1 Does your national government employ a "cascade of care" approach to monitor the numbers and proportions of people who progress through each stage of the HBV and HCV care cascades?

- ◯ Yes
- ◯ No
- ◯ Do not know

(Stages such as testing, diagnosis, linkage to care, assessment, treatment and sustained viral response.)

Click to see a definition of "cascade of care".

Additional comments: ________________________________

Sources for answers: ____________________________

4. National disease registers

4.1 Does your government or any government-related institution have a disease register for HBV infection?

◯ Yes
◯ No
◯ Do not know

Click to see a definition of "disease register".

Are subnational level (e.g., province, region) data available?

◯ Yes
◯ No
◯ Do not know

Additional comments: ________________________________

Sources for answers: ____________________________

4.2 Does your government or any government-related institution have a disease register for HCV infection?

◯ Yes
◯ No
◯ Do not know

Click to see a definition of "disease register".

Are subnational level (e.g., province, region) data available?

◯ Yes
◯ No
◯ Do not know

Additional comments: ________________________________

Sources for answers: ____________________________

4.3 Does your government or any government-related institution have a national disease register for hepatocellular carcinoma?

◯ Yes
◯ No
◯ Do not know

Click to see a definition of "disease register".

Are subnational level (e.g., province, region) data available?

◯ Yes
◯ No
◯ Do not know

Additional comments: ________________________________

Sources for answers: ____________________________

5. Availability of harm reduction services

5.1 In your country, which of the following harm reduction services are available to people who inject drugs?

	Available in all parts of the country	Available in only some parts of the country	Not available	Do not know
Needle and syringe programmes	○	○	○	○
Opioid substitution therapy	○	○	○	○
Drug consumption rooms	○	○	○	○
Other type #1 (please specify below)	○	○	○	○
Other type #2 (please specify below)	○	○	○	○
Other type #3 (please specify below)	○	○	○	○

Other type of harm reduction service (1): ______________________________

Other type of harm reduction service (2): ______________________________

Other type of harm reduction service (3): ______________________________

Additional comments: ______________________________

Sources for answers: ______________________________

5.2 In your country, which of the following harm reduction services are available in prisons?

	Available in prisons in all parts of the country	Available in prisons in only some parts of the country	Not available in prisons	Do not know
Needle and syringe programmes	○	○	○	○
Opioid substitution therapy	○	○	○	○
Other type #1 (please specify below)	○	○	○	○
Other type #2 (please specify below)	○	○	○	○
Other type #3 (please specify below)	○	○	○	○

Other type of harm reduction service available in prisons (1): ______________________________

Other type of harm reduction service available in prisons (2): ______________________________

Other type of harm reduction service available in prisons (3): ______________________________

Additional comments: ______________________

Sources for answers: ______________________

6. HBV/HCV testing/screening sites outside of hospitals

6.1 In your country, are there any HBV testing/screening sites outside of hospitals?

(Sites that are not within either inpatient or outpatient hospital facilities.)

- ◯ Yes
- ◯ No
- ◯ Do not know

What type(s) of non-hospital settings?

- ☐ General practitioner clinics
- ☐ Opioid substitution therapy clinics
- ☐ Needle and syringe programmes
- ☐ Other (please specify below)

Other type (1): ______________________________

Other type (2): ______________________________

Other type (3): ______________________________

Other type (4): ______________________________

Additional comments:

____

Sources for answers: ______________________________

6.2 In your country, are there any HCV testing/screening sites outside of hospitals?

(Sites that are not within either inpatient or outpatient hospital facilities.)

- ◯ Yes
- ◯ No
- ◯ Do not know

What type(s) of non-hospital settings?

- ☐ General practitioner clinics
- ☐ Opioid substitution therapy clinics
- ☐ Needle and syringe programmes
- ☐ Other (please specify below)

Other type (1): ______________________________

Other type (2): ______________________________

Other type (3): ______________________________

Other type (4): ______________________________

Additional comments:

____

Sources for answers: ______________________________

7. Free and anonymous HBV/HCV testing services

7.1 Is there free HBV testing targeting the general population or one or more high-risk populations in your country? (Select all that apply)

Click to see a definition of "high-risk populations".

- ☐ General population
- ☐ High-risk population(s) (please specify below)
- ☐ None of the above
- ☐ Do not know

Which high-risk population(s)? Please mark all boxes that apply.

- ☐ People who inject drugs
- ☐ Men who have sex with men
- ☐ Transgender people
- ☐ Sex workers
- ☐ Prisoners
- ☐ Migrants
- ☐ People living with HIV
- ☐ Other population(s) (please specify below)

Other population #1: ______________________________

Other population #2: ______________________________

Other population #3: ______________________________

Additional comments: ______________________________

Sources for answers: ______________________________

7.2 Is there anonymous HBV testing targeting the general population or one or more high-risk populations in your country? (Select all that apply)

Click to see a definition of "high-risk populations".

- ☐ General population
- ☐ High-risk population(s) (please specify below)
- ☐ None of the above
- ☐ Do not know

Which high-risk population(s)? Please mark all boxes that apply.

- ☐ People who inject drugs
- ☐ Men who have sex with men
- ☐ Transgender people
- ☐ Sex workers
- ☐ Prisoners
- ☐ Migrants
- ☐ People living with HIV
- ☐ Other population(s) (please specify below)

Other population #1: ______________________________

Other population #2: ______________________________

Other population #3: ______________________________

Additional comments: ______________________________

Sources for answers: ______________________________

7.3 Is there free HCV testing targeting the general population or one or more high-risk populations in your country? (Select all that apply)

Click to see a definition of "high-risk populations".

- ☐ General population
- ☐ High-risk population(s) (please specify below)
- ☐ None of the above
- ☐ Do not know

Which high-risk population(s)? Please mark all boxes that apply.

- ☐ People who inject drugs
- ☐ Men who have sex with men
- ☐ Transgender people
- ☐ Sex workers
- ☐ Prisoners
- ☐ Migrants
- ☐ People living with HIV
- ☐ Other population(s) (please specify below)

Other population #1: ______________________________

Other population #2: ______________________________

Other population #3: ______________________________

Additional comments: ______________________________

Sources for answers: ______________________________

7.4 Is there anonymous HCV testing targeting the general population or one or more high-risk populations in your country? (Select all that apply)

Click to see a definition of "high-risk populations".

- ☐ General population
- ☐ High-risk population(s) (please specify below)
- ☐ None of the above
- ☐ Do not know

Which high-risk population(s)? Please mark all boxes that apply.

- ☐ People who inject drugs
- ☐ Men who have sex with men
- ☐ Transgender people
- ☐ Sex workers
- ☐ Prisoners
- ☐ Migrants
- ☐ People living with HIV
- ☐ Other population(s) (please specify below)

Other population #1: ______________________________

Other population #2: ______________________________

Other population #3: ______________________________

Additional comments: ______________________________

Sources for answers: ______________________________

8. Liver enzyme and/or risk assessment for HBV/HCV in routine medical check-ups

8.1 Is liver enzyme testing included in routine primary care medical check-ups in your country?

◯ Yes
◯ No
◯ Do not know

8.2 Is risk assessment for HBV/HCV included in routine primary care medical check-ups in your country?

◯ Yes
◯ No
◯ Do not know

Click to see the definition of "risk assessment"

Additional comments: ______________________

Sources for answers: ______________________

9. Treatment of HCV patients in non-hospital settings

9.1 Do any HCV patients in your country have the option of being treated in non-hospital settings?

(Settings that are not within either inpatient or outpatient hospital facilities.)

○ Yes
○ No
○ Do not know

What type(s) of non-hospital setting?

☐ General practitioner clinics
☐ Opioid substitution therapy clinics
☐ Needle and syringe programmes
☐ Other (please specify below)

Other type, please specify (1): ____________________________

Other type, please specify (2): ____________________________

Other type, please specify (3): ____________________________

Additional comments: ____________________________

Sources for answers: ____________________________

10. HBV and HCV treatment provided in prisons

10.1 Is HBV treatment provided in prisons in your country?

- ◯ Yes
- ◯ No
- ◯ Do not know

If yes, what percentage of prisons provide HBV treatment?

- ◯ Do not know
- ◯ 0-9%
- ◯ 10-19%
- ◯ 20-29%
- ◯ 30-39%
- ◯ 40-49%
- ◯ 50-59%
- ◯ 60-69%
- ◯ 70-79%
- ◯ 80-89%
- ◯ 90-99%
- ◯ 100%

10.2 Is HCV treatment provided in prisons in your country?

- ◯ Yes
- ◯ No
- ◯ Do not know

If yes, what percentage of prisons provide HCV treatment?

- ◯ Do not know
- ◯ 0-9%
- ◯ 10-19%
- ◯ 20-29%
- ◯ 30-39%
- ◯ 40-49%
- ◯ 50-59%
- ◯ 60-69%
- ◯ 70-79%
- ◯ 80-89%
- ◯ 90-99%
- ◯ 100%

Additional comments: ________________________________

Sources for answers: __________________________

11. Restrictions on access to direct-acting antivirals for the treatment of HCV infection

11.1 In practice, what restrictions are there on access to direct-acting antivirals for the treatment of HCV infection in your country?

Please choose all answers that apply.

- ☐ None
- ☐ Fibrosis level: only patients above a certain fibrosis level are eligible for treatment
- ☐ Quotas: only a limited number of patients can be treated within a certain time period or a certain geographic area
- ☐ Alcohol use: people who currently drink alcohol are not treated
- ☐ Injecting drug use: people who injected drugs in the past are not treated, even if they are not currently injecting drugs
- ☐ Injecting drug use: people who injected drugs in the past are not treated, even if they are not currently injecting drugs, unless they are receiving opioid substitution therapy
- ☐ Injecting drug use: people who are currently injecting drugs are not treated
- ☐ Injecting drug use: people who are currently injecting drugs are not treated unless they are receiving opioid substitution therapy
- ☐ Injecting drug use: people who injected drugs in the past are only treated if they have abstained from injecting drugs for a specified period of time
- ☐ Other restrictions (please describe below)
- ☐ Do not know

Other restriction, please describe (1): ______________________________

Other restriction, please describe (2): ______________________________

Other restriction, please describe (3): ______________________________

Additional comments:

____

Sources for answers: ______________________________

NOTICE
ONLY click on the "Submit" button below if you have completed your work on the survey.

If instead you wish to save your work and continue responding to the survey at a later time, please click on the "Save & Return Later" button.
