# Supplementary material for: Hep‐CORE: a cross‐sectional study of the viral hepatitis policy environment reported by patient groups in 25 European countries in 2016 and 2017
Source: J Int AIDS Soc. 2018 Apr 10;21(Suppl Suppl 2):e25052. doi: 10.1002/jia2.25052 (PMC5978657; doi:10.1002/jia2.25052)
Supplement: Supplementary file 3 — Additional File 3. Detailed comparative data on national coordination, monitoring, prevention, screening and treatment for Hep‐CORE 2016 and 2017 (N = 25 countries). [file JIA2-21-e25052-s003.docx]

**Detailed comparative data on national coordination, monitoring, prevention, screening and treatment for Hep-CORE 2016 and 2017 (N = 25 countries).**

|  | **2016** | | | | **2017** | | | | | | | | | |
| --- | --- | --- | --- | --- | --- | --- | --- | --- | --- | --- | --- | --- | --- | --- |
|  | Yes | | No | Do not know | Yes | | | | | | No | | | Do not know |
| ***National coordination*** |  | |  |  | |  | | | | |  | | |  |
| Written national HBV strategy | 8 (32%)  France, Germany, Italy, Romania, Slovakia, Slovenia, Turkey, Ukraine | | 17 (68%)  Austria, Belgium, Bosnia and Herzegovina, Bulgaria, Croatia, Denmark, Finland, Greece, Hungary, Macedonia, Netherlands, Poland, Portugal, Serbia, Spain, Sweden, United Kingdom | 0 | 7 (28%)  France, Germany, Italy, Netherlands, Slovenia, Turkey, Ukraine | | | | | | 18 (72%)  Austria, Belgium, Bosnia and Herzegovina, Bulgaria, Croatia, Denmark, Finland, Greece, Hungary, Macedonia, Poland, Portugal, Romania, Serbia, Slovakia, Spain, Sweden, United Kingdom | | | 0 |
| Written national HCV strategy | 11 (44%)  Belgium, France, Germany, Italy, Romania, Slovakia, Slovenia, Spain, Turkey, Ukraine, United Kingdom | | 14 (56%)  Austria, Bosnia and Herzegovina, Bulgaria, Croatia, Denmark, Finland, Greece, Hungary, Macedonia, Netherlands, Poland, Portugal, Serbia, Sweden | 0 | 12 (48%)  Belgium, Finland, France, Germany, Greece, Italy, Portugal, Slovenia, Spain, Turkey, Ukraine, United Kingdom | | | | | | 13 (52%)  Austria, Bosnia and Herzegovina, Bulgaria, Croatia, Denmark, Hungary, Macedonia, Netherlands, Poland, Romania, Serbia, Slovakia, Sweden | | | 0 |
| Government collaborates with in-country civil society groups to plan and carry out its viral hepatitis programme^a^ | 13 (52%)  Austria, Bulgaria, Croatia, Germany, Greece, Italy, Macedonia, Portugal, Slovenia, Sweden, Turkey, Ukraine, United Kingdom | | 9 (36%)  Belgium, Bosnia and Herzegovina, Denmark, Finland Hungary, Netherlands, Serbia, Slovakia, Spain | 3 (12%)  France, Poland, Romania | 18^b^ (75%)  Belgium, Bulgaria, Croatia, Finland, France, Germany, Greece, Italy, Macedonia, Netherlands, Poland, Portugal, Romania, Slovenia, Sweden, Turkey, Ukraine, United Kingdom | | | | | | 5^b^ (21%)  Austria, Bosnia and Herzegovina, Denmark, Hungary, Serbia, | | | 1^b^ (4%)  Slovakia |
| ***Monitoring*** |  | |  |  |  | | | | | |  | | |  |
| Government or government-related institution has national HBV disease register | 9 (36%)  Belgium, Denmark, Finland, Germany, Hungary, Slovenia, Sweden, Turkey, United Kingdom | | 16 (64%)  Austria, Bosnia and Herzegovina, Bulgaria, Croatia, France, Greece, Italy, Macedonia, Netherlands, Poland, Portugal, Romania, Serbia, Slovakia, Spain, Ukraine | 0 | 9 (36%)  Austria, Finland, France, Greece, Slovakia, Slovenia, Turkey, Ukraine, United Kingdom | | | | | | 15 (60%)  Belgium, Bosnia and Herzegovina, Bulgaria, Croatia, Denmark, Finland, Greece, Italy, Macedonia, Netherlands, Poland, Portugal, Romania, Serbia, Spain | | | 1 (4%)  Sweden |
| Government or government-related institution has national HCV disease register | 11 (44%)  Austria, Denmark, Finland, Germany, Greece, Hungary, Portugal, Slovenia, Sweden, Turkey, United Kingdom | | 14 (56%)  Belgium, Bosnia and Herzegovina, Bulgaria, Croatia, France, Italy, Macedonia, Netherlands, Poland, Romania, Serbia, Slovakia, Spain, Ukraine | 0 | 11 (44%)  Austria, Germany, Greece, Hungary, Portugal, Slovakia, Slovenia, Sweden, Turkey, Ukraine, United Kingdom | | | | | | 14 (56%)  Belgium, Bosnia and Herzegovina, Bulgaria, Croatia, Denmark, Finland, France, Italy, Macedonia, Netherlands, Poland, Romania, Serbia, Spain | | | 0 |
| ***Prevention*** |  | |  |  |  | | | | | |  | | |  |
| Harm reduction services available: Needle and syringe programmes (All: *available* *in all parts of country, Some: available in some parts of country)* | **All:**  10  (40%)  Austria, Belgium, Denmark, Greece, Netherlands, Portugal, Slovenia, Spain, Turkey, United Kingdom | **Some:**  10  (40%)  Bulgaria, Croatia, Finland, France, Germany, Macedonia, Romania, Slovakia, Sweden, Ukraine | 4  (16%)  Bosnia and Herzegovina, Hungary, Italy, Serbia | 1  (4%)  Poland | **All:**  8  (32%)  Austria, Belgium, France, Netherlands, Portugal, Slovakia, Slovenia, United Kingdom | | | **Some:** 12  (48%)  Bulgaria, Croatia, Finland, Germany, Greece, Italy, Macedonia, Poland, Romania, Spain, Sweden, Ukraine | | | 3  (12%)  Bosnia and Herzegovina, Hungary, Serbia | | | 2  (8%)  Denmark, Turkey |
| Harm reduction services available: Opioid substitution therapy (All: *available in all parts of country, Some: available in some parts of country)* | **All:**  22  (88%)  Austria, Belgium, Bosnia and Herzegovina, Bulgaria, Croatia, Finland, France, Germany, Greece, Hungary, Italy, Macedonia, Netherlands, Portugal, Serbia, Slovakia, Slovenia, Spain, Sweden, Turkey, Ukraine, United Kingdom | **Some:**  1  (4%)  Denmark | 0 | 2  (8%)  Poland, Romania | **All:**  20  (80%)  Austria, Belgium, Croatia, France, Germany, Greece, Hungary, Italy, Macedonia, Netherlands, Portugal, Romania, Serbia, Slovakia, Slovenia, Spain, Sweden, Turkey, Ukraine, United Kingdom | | **Some:**  4  (16%)  Bosnia and Herzegovina, Bulgaria, Finland, Poland | | | 0 | | | 1  (4%)  Denmark | |
| Harm reduction services available: Drug consumption rooms (All: *available in all parts of country, Some: available in some parts of country)* | **All:**  2  (8%)  Denmark, Germany | **Some:**  2  (8%)  Netherlands, Spain | 17  (68%)  Austria, Belgium, Bosnia and Herzegovina, Bulgaria, Croatia, Finland, France, Greece, Italy, Portugal, Serbia, Slovakia, Slovenia, Sweden, Turkey, Ukraine, United Kingdom | 4  (16%)  Hungary, Macedonia, Poland, Romania | **All:**  1  (4%)  Austria | | | | **Some:**  5  (20%)  Denmark, France, Germany, Slovenia, Spain | | 16  (64%)  Belgium, Bosnia and Herzegovina, Bulgaria, Croatia, Finland, Greece, Hungary, Italy, Macedonia, Poland, Portugal, Romania, Serbia, Slovakia, Ukraine, United Kingdom | 3  (12%)  Netherlands, Sweden, Turkey | | |
| ***Screening*** |  | |  |  |  | | | | | |  |  | | |
| Risk assessment for HBV/HCV included in routine medical check-ups | 5  (20%)  Austria, Denmark, Hungary, Italy, Turkey | | 20  (80%)  Belgium, Bosnia and Herzegovina, Bulgaria, Croatia, Finland, France, Germany, Greece, Macedonia, Netherlands, Poland, Portugal, Romania, Serbia, Slovakia, Slovenia, Spain, Sweden, Ukraine, United Kingdom | 0 | 6  (24%)  Bosnia and Herzegovina, Bulgaria, Denmark, Italy, Macedonia, Ukraine | | | | | | 18  (72%)  Austria, Belgium, Croatia, France, Germany, Greece, Hungary, Netherlands, Poland, Portugal, Romania, Serbia, Slovakia, Slovenia, Spain, Sweden, Turkey, United Kingdom | 1  (4%)  Finland | | |
| Liver enzyme testing included in routine medical check-ups | 17  (68%)  Austria, Belgium, Bosnia and Herzegovina, Croatia, Denmark, Finland, France, Greece, Hungary, Italy, Macedonia, Romania, Slovakia, Slovenia, Turkey, Ukraine, United Kingdom | | 8  (32%)  Bulgaria, Germany, Netherlands, Poland, Portugal, Serbia, Spain, Sweden | 0 | 14  (56%)  Austria, Belgium, Bosnia and Herzegovina, Croatia, France, Hungary, Italy, Macedonia, Poland, Romania, Slovenia, Slovakia, Turkey, Ukraine | | | | | | 10  (40%)  Bulgaria, Finland, Germany, Greece, Netherlands, Portugal, Serbia, Spain, Sweden, United Kingdom | 1  (4%)  Denmark | | |
| ***Treatment*** |  | |  |  |  | | | | | |  |  | | |
| HBV treatment provided in prisons | 18^c^  (75%)  Austria, Belgium, Bulgaria, Denmark, Finland, France, Germany, Hungary, Italy, Netherlands, Poland, Portugal, Serbia, Slovakia, Slovenia, Spain, Sweden, United Kingdom | | 5^c^  (21%)  Bosnia and Herzegovina, Croatia, Greece, Macedonia, Ukraine | 1^c^  (4%)  Turkey | 19  (76%)  Austria, Belgium, Bulgaria, Denmark, Finland, France, Germany, Hungary, Italy, Netherlands, Portugal, Serbia, Slovakia, Slovenia, Spain, Sweden, Turkey, Ukraine, United Kingdom | | | | | | 5  (20%)  Bosnia and Herzegovina, Croatia, Greece, Macedonia, Poland | 1  (4%)  Denmark | | |
| HCV patients have option to be treated in non-hospital settings^d^ | 5  (20%)  France, Germany, Romania, Turkey, United Kingdom | | 20  (80%)  Austria, Belgium, Bosnia and Herzegovina, Bulgaria, Croatia, Denmark, Finland, Greece, Hungary, Italy, Macedonia, Netherlands, Poland, Portugal, Serbia, Slovakia, Slovenia, Spain, Sweden, Ukraine | 0 | 5  (20%)  France, Germany, Turkey, Ukraine, United Kingdom | | | | | | 20  (80%)  Austria, Belgium, Bosnia and Herzegovina, Bulgaria, Croatia, Denmark, Finland, Greece, Hungary, Italy, Macedonia, Netherlands, Poland, Portugal, Romania, Serbia, Slovakia, Slovenia, Spain, Sweden | 0 | | |
| HBV = hepatitis B virus, HCV = hepatitis C virus | | | | | | | | | | | | | | |
| a. Survey respondents were advised that the following are not considered in-country civil society groups: United Nations agencies, international NGOs, government ministries, university programmes and military programmes. | | | | | | | | | | | | | | |
| b. 2017 responses to this question total 24 instead of 25 because there was one non-response. | | | | | | | | | | | | | | |
| c. 2016 responses to this question total 24 instead of 25 because there was one non-response. | | | | | | | | | | | | | | |
| d. Settings that are not within either inpatient or outpatient hospital facilities. | | | | | | | | | | | | | | |

**Reported availability of free and anonymous testing services in study countries (N=25)**

|  | **2016** | **2017** |
| --- | --- | --- |
| Free HBV testing for the general population | 9 (36%)  Croatia, Denmark, France, Macedonia, Netherlands, Serbia, Slovenia, Sweden, Turkey | 12 (48%)  Croatia, France, Germany, Greece, Macedonia, Poland, Serbia, Slovakia, Slovenia, Spain, Sweden, United Kingdom |
| Free HBV testing for high-risk populations | 17 (68%)  Croatia, Denmark, Finland, France, Germany, Hungary, Italy, Macedonia, Netherlands, Portugal, Serbia, Slovakia, Slovenia, Sweden, Turkey, Ukraine, United Kingdom | 17 (68%)  Bulgaria, Croatia, Finland, Germany, Greece, Hungary, Italy, Macedonia, Portugal, Romania, Serbia, Slovakia, Slovenia, Spain, Sweden, Ukraine, United Kingdom |
| Anonymous HBV testing for the general population | 6 (24%)  Croatia, France, Macedonia, Slovenia, Sweden, Turkey | 12 (48%)  Bulgaria, Croatia, France, Germany, Greece, Macedonia, Serbia, Slovakia, Slovenia, Spain, Sweden, Ukraine |
| Anonymous HBV testing for high-risk populations | 11 (44%)  Croatia, France, Italy, Macedonia, Netherlands, Portugal, Slovakia, Slovenia, Sweden, Turkey, United Kingdom | 13 (52%)  Bulgaria, Croatia, Germany, Greece, Hungary, Macedonia, Portugal, Serbia, Slovakia, Slovenia, Spain, Sweden, Ukraine |
| Free HCV testing for the general population | 9 (36%)  Croatia, Denmark, France, Macedonia, Netherlands, Serbia, Slovenia, Sweden, Turkey | 12 (48%)  Bulgaria, Croatia, France, Germany, Greece, Macedonia, Portugal, Serbia, Slovakia, Slovenia, Spain, Sweden |
| Free HCV testing for high-risk populations | 16 (64%)  Croatia, Denmark, Finland, France, Germany, Hungary, Italy, Macedonia, Netherlands, Portugal, Serbia, Slovakia, Slovenia, Sweden, Turkey, Ukraine | 17 (68%)  Bulgaria, Croatia, Finland, Germany, Greece, Hungary, Italy, Macedonia, Portugal, Romania, Serbia, Slovakia, Slovenia, Spain, Sweden, Ukraine, United Kingdom |
| Anonymous HCV testing for the general population | 6 (24%)  Croatia, France, Macedonia, Slovenia, Spain, Turkey | 11 (44%)  Bulgaria, Croatia, France, Germany, Greece, Macedonia, Serbia, Slovakia, Slovenia, Spain, Sweden |
| Anonymous HCV testing for high-risk populations | 10 (40%)  Croatia, France, Italy, Macedonia, Netherlands, Portugal, Slovakia, Slovenia, Sweden, Turkey | 13 (52%)  Bulgaria, Croatia, Germany, Greece, Hungary, Macedonia, Portugal, Serbia, Slovakia, Slovenia, Spain, Sweden, Ukraine |

**Reported restrictions on access to direct-acting antivirals for the treatment of HCV in study countries (N=25)**

|  | **2016** | **2017** |
| --- | --- | --- |
| None | 3 (12%)  France, Netherlands, Portugal | 7 (28%)  Austria, Bulgaria, France, Italy, Netherlands, Portugal, Turkey |
| Fibrosis level | 18 (72%)  Austria, Belgium, Bulgaria, Croatia, Denmark, Finland, Greece, Hungary, Italy, Macedonia, Romania, Slovakia, Slovenia, Spain, Sweden, Turkey, Ukraine, United Kingdom | 13 (52%)  Belgium, Croatia, Denmark, Finland, Greece, Hungary, Macedonia, Romania, Slovakia, Slovenia, Spain, Sweden, Ukraine |
| Quotas | 7 (28%)  Croatia, Denmark, Finland, Poland, Slovakia, Spain, United Kingdom | 8 (32%)  Belgium, Bosnia and Herzegovina, Croatia, Finland, Romania, Slovakia, Ukraine, United Kingdom |
| Alcohol use | 8 (32%)  Austria, Bulgaria, Croatia, Germany, Hungary, Spain, Sweden, Ukraine | 5 (20%)  Croatia, Denmark, Finland, Romania, Ukraine |
| People currently injecting drugs not treated | 13 (52%)  Austria, Bulgaria, Croatia, Finland, Germany, Hungary, Macedonia, Poland, Romania, Slovakia, Spain, Sweden, Ukraine | 8 (32%)  Belgium, Croatia, Finland, Hungary, Macedonia, Romania, Slovakia, Ukraine |
| People currently injecting drugs not treated unless receiving treatment with OST^a^ | 6 (24%)  Austria, Bulgaria, Denmark, Germany, Romania, Slovakia | 6 (24%)  Austria, Croatia, Finland, Romania, Slovakia, Ukraine |
| People who injected drugs in past only treated if abstained from injecting for specified time | 8 (32%)  Austria, Bulgaria, Croatia, Denmark, Finland, Slovakia, Spain, Ukraine | 6 (24%)  Austria, Belgium, Croatia, Finland, Slovakia, Ukraine |
| People injecting in past not treated, even if not currently injecting drugs | 1 (4%)  Croatia | 2 (8%)  Denmark, Finland |
| People who injected drugs in the past not treated, even if not currently injecting drugs, unless receiving treatment with OST^a^ | n/a | 2 (8%)  Denmark, Finland |
| Other | 4 (16%)  Belgium, Croatia, Poland, Romania  (*Other* *included: gastroenterologist at university hospital prescription only, people younger than 18 years, people older than 70 years, patients with genotype 3)* | 5 (20%)  Croatia, Germany, Poland, Romania, United Kingdom  (*Other* *included: people younger than 18 years, patients with genotype 3, medical insurance limitations, retreatment)* |

a. This is one of two 2017 response options that were reflected in a single response option in 2016. The 2016 option read, “People currently injecting drugs or who injected drugs in the past not treated unless receiving OST”.
